# The theory of planned behavior and dietary behaviors in competitive women bodybuilders

**DOI:** 10.1186/s12889-023-16568-w

**Published:** 2023-09-04

**Authors:** John E. Haubenstricker, Jerry W. Lee, Gina Segovia-Siapco, Ernesto Medina

**Affiliations:** Loma Linda University, School of Public Health, 24951 Circle Drive, Loma Linda, CA 92354 USA

**Keywords:** Women, Bodybuilder, Dietary supplement, Protein, Competitor, Theory of Planned Behavior

## Abstract

**Background:**

Women bodybuilders build their ideal physique by manipulating their diet, supplement, and exercise regimens to extreme levels. Excess protein intake and dietary supplement use is ubiquitous in women bodybuilders preparing for a competition, i.e., in-season competitors, however the impetus for these two dietary behaviors are relatively unknown. The Theory of Planned Behavior (TPB) has been used to explain dietary behaviors. The purpose of the study was to examine how the TPB can explain protein intake and dietary supplement use in in-season competitors.

**Methods:**

Using a cross-sectional design, an online questionnaire was developed, validated, and administered to collect dietary supplement use, TPB variables, and other measures from 112 in-season competitors. Protein intake was assessed using multiple 24-h dietary recalls. Associations between TPB and protein intake and dietary supplement use were determined with multiple regression analysis while adjusting for confounders.

**Results:**

For protein intake: attitude, subjective norm, and perceived behavioral control explained 8% of the variance in intention; subjective norm independently predicted intention. Behavioral beliefs predicted attitude; subjective norm was predicted by trainer/coach, workout partners, and social media influencers. For dietary supplement use: intention explained 5% of the variance in dietary supplement use; attitude, subjective norm, and perceived behavioral control together explained 38% of the variance in intention. Attitudes towards dietary supplements use were predicted by five factors (not a waste of money, help improve physique, sustain energy levels, provide enough calories, help with recovery). Primary determinants of subjective norm were fellow competitors, social media influencers, and trainer/coach. Perceived behavioral control was predicted by three factors (ease of purchase, affordability to purchase, availability to purchase).

**Conclusions:**

TPB predicted dietary supplement use in women bodybuilders during in-season but there was little evidence for the prediction of protein intake using the TPB. Health professionals should develop effective interventions using strategies that align health education messages with in-season competitors’ outcome beliefs and collaborate with their referent others to influence safer and effective dietary supplement use.

**Supplementary Information:**

The online version contains supplementary material available at 10.1186/s12889-023-16568-w.

## Background

Women’s bodybuilding is a sport in which competitors are critiqued on their level of muscularity, leanness, symmetry, and proportion to achieve their desired physique. The precise mix of these characteristics are determined by the different competition divisions, i.e., bikini, bodybuilding, figure, fitness, physique, or wellness. To obtain the desired physique required for competition, diet, supplement, and exercise regimes are manipulated to extreme levels.

Women bodybuilders who are preparing for a competition, i.e., in-season competitors, typically consume copious amounts of protein (150–204 g/d; 2.5–3.5 g/kg of body wt [kg BW]/d) and a myriad of dietary supplements [1–6]. Most of the studies, i.e., five out of the six studies, assessing protein intake in in-season competitors were above the recommended intakes levels (1.8–2.7 g/kg BW/d) [7]. All six studies also reported dietary supplement use by 100% of the competitors during the in-season [1–6]. In-season competitors used between 3 to 21 different supplements with some of the most common supplements being protein powders, multivitamin/multimineral, individual vitamins/minerals, creatine, branched chain amino acids, and energy drinks/fat burners. The literature has indicated that dietary supplements can be contaminated and associated with adverse health events [8–10]. Although excess protein intake and dietary supplement use is ubiquitous in competitors during the in-season, to the authors’ knowledge, the impetus for these two dietary behaviors are relatively unknown in these athletes.

The Theory of Planned Behavior (TPB) was developed to help explain and predict human behaviors [11]. According to Ajzen [12], human behaviors are directed by three main beliefs—behavioral beliefs (the likely outcomes of the behavior), normative beliefs (the expectations of important others), and control beliefs (the factors that may affect behavior execution). The aggregate of the mathematical product of each set of behavioral beliefs (each salient behavioral belief and associated subjective outcome evaluation), normative beliefs (each salient injunctive and descriptive normative belief), and control beliefs (each salient control factor in conjunction with the perceived power for each control factor) results in composite belief indices that are directly proportional to attitude, subjective norm, and perceived behavioral control toward the behavior, respectively.

The TPB posits that attitude (the personal valuation of the behavior), subjective norm (the social pressure we perceive from important others), and perceived behavioral control (our perception of how easy or hard it is to perform the behavior) predict intentions to perform a behavior [11]. Each of the three TPB constructs are defined by two components: 1) the instrumental (the usefulness of the behavior) and experiential (the feeling toward the behavior) for attitude; 2) the injunctive (what we believe important others think we should do) and descriptive (our beliefs of what important others do) for subjective norm; and 3) the capacity (whether we believe we can perform the behavior) and autonomy (how much control we have over the behavior) for perceived behavioral control [13]. Additionally, perceived behavioral control can also predict the execution of a behavior to the extent that perceived control matches actual control [13]. Together intention and perceived behavioral control account for the execution of the behavior [13]. In essence, the intent to perform the behavior, and thus the execution of the behavior is determined by a positive evaluation of the behavior, the belief that important others think we should perform the behavior and do it themselves, and the degree of perceived control over the behavior.

Research indicates that the constructs of the TPB predict dietary behaviors in women and athletes. In a 2015 systematic review, associations between the TPB constructs and discrete dietary behaviors, e.g., fruits, vegetables, had medium to large (0.27–0.54) pooled effect sizes (r_+_) in studies examining Western populations that were predominately female [14]. One study examining the association between the TPB and dietary supplement consumption in National Collegiate Athletic Association Division 1 female student athletes found the TPB constructs, i.e., subjective norms, perceived behavioral control, and attitudes, were able to explain 64–66% of variance in intent to consume a dietary supplement [15]. Similarly, the three TPB constructs were also able to explain 42–43% of the dietary supplement behavior [15]. Although research has suggested diet and dietary supplement intake are influenced by salient normative beliefs shaping the subjective norms in in-season competitors, there has not been a formal investigation using the TPB [16]. From a public health perspective, it is important to understand the causal processes related to competitive women bodybuilder’s potentially excessive dietary protein intake and dangerous dietary supplement behaviors due to increased utilization of those dietary behaviors by the general public and military personnel [17–19]. Understanding the factors that lead to in-season competitors’ dietary protein intake and use of dietary supplements will help health educators develop strategies that can shape their food and supplement choices and those of bodybuilding adherents.

**Fig. 1:**
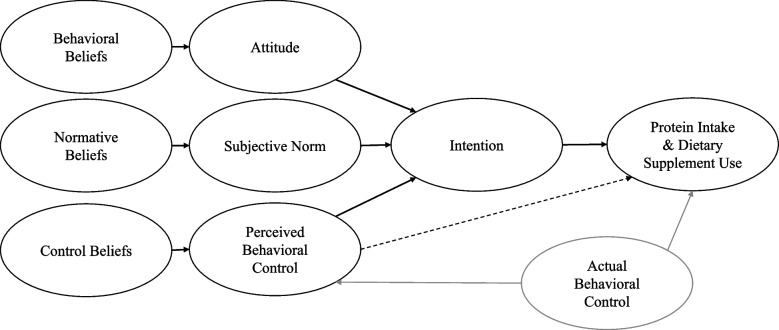
The theoretical framework illustrating the TPBs proposed influence on protein intake and supplement use

Thus, the aim of this study is to examine how the TPB can enhance our understanding of in-season competitors’ dietary protein and dietary supplement behaviors. To the authors’ knowledge the predictors of protein and dietary supplement intake using the TPB have not been examined in in-season competitors. Figure 1 shows the theoretical framework on how the TPB beliefs and constructs are proposed to influence protein intake and dietary supplement use among competitors during the in-season.

## Methods

### Study design and participants

The study employed a cross-sectional design using a self-constructed online questionnaire and a dietary assessment tool for collecting data on in-season competitors. In-season competitors were recruited via purposive and snowball sampling from social media platforms, known physique coaches, professional colleagues, local gyms, and known in-season competitors throughout the US. To participate in the study, eligible participants had to be willing to complete the online questionnaire and four dietary recalls, be currently preparing for a bodybuilding competition, (i.e., in-season), in one of the physique divisions (i.e., bodybuilding, bikini, figure, fitness, physique, or wellness), be at least 18 years old and female, fluent in English, currently reside in the US, and have internet access. Data collection took place between July and November 2020. Interested participants were sent a link to the study website to complete an online screener via Qualtrics [20] to see whether they met the inclusion criteria to participate in the study. Those who qualified to participate in the study received a link to the online questionnaire via Qualtrics [20] and were asked to provide consent online prior to responding to the questionnaire. Participants who completed the questionnaire were emailed or texted a unique username and password to complete the four 24-h dietary recalls via Automated Self-Administered 24-Hour (ASA24®) Dietary Assessment Tool, version (2020), developed by the National Cancer Institute, Bethesda, MD [21]. Reminder text messages and emails were sent to ensure participants completed their dietary recalls. Participants were incentivized ($100) to complete the online questionnaire and four 24-h recalls.

G*Power 3.1.9.2 was used to determine the sample size for this study. Using multiple linear regression with a total of 15 predictor variables and covariates in a fixed model, and setting power at 80%, a medium effect size (*f *^*2*^ = 0.11), an α = 0.05 for significance, and accounting for 10% missing data, approximately 114 participants were needed to detect a 10% increase in the variance explained by the predictor [22].

All procedures were conducted in accordance with the applicable guidelines and regulations, and study reporting conformed with the STROBE Statement for cross-sectional studies. All protocols, marketing materials, and study website were approved by Loma Linda Institutional Review Board (IRB# 5180399). This study is covered by a Certificate of Confidentiality from the National Institutes of Health (#CC-OD-20–527).

### Instrument development and validation

An online questionnaire was developed to assess dietary supplement use, sociodemographic variables, bodybuilder variables, the TPB beliefs, i.e., behavioral, normative, and control beliefs, and their underlying constructs, i.e., attitude, subjective norm, perceived behavioral control and intention, in in-season competitors. Items for dietary supplement use, sociodemographic and bodybuilder information were created based on the limitations discussed in a systematic review [23], a sample dietary assessment questionnaire for bodybuilders [1], and a focus group. A 90-min in-person focus group of nine women bodybuilding competitors who met the inclusion criteria for the study helped identify and provide insight into the relevant dietary supplements, sociodemographic, and bodybuilder characteristics in in-season competitors. Details of the focus group methodology and analyses had been described previously [24]. Before incorporation into the final online questionnaire, items for the TPB beliefs section were separately developed and administered to 21 women bodybuilding competitors. Then, a content analysis was performed [25] to acquire a list of modal salient beliefs for each behavior to design the protein and dietary supplement beliefs items used in the final questionnaire.

The validation of the online questionnaire included assessing the reliability and validity of the TPB constructs; pilot-testing and content validation of the sociodemographic, bodybuilder, and dietary supplement variables (i.e., all non-TPB items); and cognitive testing of the final questionnaire. A confirmatory analysis was performed to ensure valid and reliable items were selected to assess the TPB constructs. A high degree of internal consistency was sought to ensure the TPB items selected assessed each of the constructs, [25] i.e., the attitude toward the behavior, subjective norm, perceived behavioral control and intention, for both protein intake and dietary supplement use. The Cronbach α values for the protein intake TPB items are as follows: attitude = 0.820 (strong reliability), subjective norms = 0.918 (strong reliability), perceived behavioral control = 0.717 (strong reliability), and intention = 0.880 (strong reliability). The Cronbach α values for the dietary supplement use TPB items are as follows: attitude = 0.645 (moderate reliability), subjective norms = 0.871 (strong reliability), perceived behavioral control = 0.694 (moderate reliability), and intention = 1.0 (strong reliability). Since the Cronbach α values for all the TPB items ranged from 0.65 to 1.0, indicating moderate to strong reliability, all items were retained in the final questionnaire.

Content validity for the non-TPB items was assessed using item-level and scale-level content validity indices (CVI) and the multi-rater kappa coefficient. Content validation experts [26] included individuals with subject matter expertise in sport nutrition, i.e., sports dietitians, from across the U.S. These experts were recruited via purposive and snowball sampling from known dietetic professionals and colleagues. The first iteration of content validation assessed the initial item-level CVI, and a second iteration evaluated the item-level CVI and the scale-level CVI. For the item-level CVI, each rater/expert rated each item for its relevance to the underlying construct using a four-point ordinal scale (1 = not relevant, 2 = somewhat relevant, 3 = quite relevant, and 4 = highly relevant), which was later collapsed into a binary scale (1 = quite / highly relevant vs. 0 = not / somewhat relevant). Afterwards, item-level CVI was calculated as the proportion of raters/experts rating the item as relevant on this binary scale.

To adjust for inter-rater agreement by chance, the modified multi-rater kappa was calculated using the modified kappa formula [24]. Scale level CVI was calculated as the average of all item-level CVIs for items in a given construct, e.g., sociodemographic or bodybuilder characteristics, by all raters/experts [24]. The first iteration of the questionnaire revealed 15 questions with an item-level CVI < 0.78. The second iteration revealed a scale-level CVI of 0.92 or higher and a modified kappa rating of excellent for all scales [27, 28].

The cognitive method of retrospective probing was performed to study the way women bodybuilding competitors process and respond to the non-TPB items in the online questionnaire [29]. This method enabled women bodybuilding competitors to successfully navigate the online questionnaire without interruption and allowed the researcher the ability to observe any technical difficulties that arose during the completion of the questionnaire [30]. Every competitor completed the questionnaire online prior to the retrospective probing. Retrospective probing was conducted on 2–6 women bodybuilding competitors at each 2-h cognitive testing session. This method required a total of 20 women bodybuilding competitors, i.e., bikini, figure, physique, and wellness, detecting at least 50% of the more serious problems affecting survey measurement error 50–90% of the time for our questionnaire [31]. The interviewer’s notes and audio recording transcription to the retrospective probing question’s responses were aggregated and summarized for each question to revise the questionnaire [30]. The revised questionnaire was compared to the previous version to demonstrate the revision had either fewer problems or eliminated problems within the questionnaire [29]. A total of five separate retrospective probing sessions was conducted to achieve saturation without discovering new high-impact problems [32].

The final validated online questionnaire has four main sections, which includes sociodemographics, bodybuilder, supplements, and the TPB—with a total of 549 items plus open-ended questions in the dietary supplements section. Items are primarily multiple-choice, and a few are open-ended. The TPB section has a 7-point bipolar adjective scale.

### Measurement of TPB variables

The validated online questionnaire developed for this study was used to measure the TPB variables. The questionnaire items for attitude (five items), subjective norm (six item), perceived behavioral control (four items) and intention (three items) for both protein intake and dietary supplement use can be found in Supplementary Table 1 (see Additional file 1) and Supplementary Table 2 (see Additional file 2), respectively. Intention was the predictor of protein intake and dietary supplement use. The predictors for intention were attitude, subjective norm, and perceived behavioral control. In turn, behavioral beliefs, normative beliefs, and control beliefs were used to predict attitude, subjective norm, and perceived behavioral control. Attitude, subjective norm, and perceived behavioral control, for each outcome variable, i.e., protein intake and dietary supplement use, was assessed using the methods outlined in Ajzen [25]. In addition, the prediction of: (a) attitude by the product of behavioral beliefs and outcome evaluation, (b) subjective norm by the product of normative beliefs and intent to comply, and (c) perceived behavioral control by the product of control beliefs and perceived power was assessed using the methods outlined in Ajzen [25].

### Measurement of dietary supplement use

In the dietary supplement use section of the validated online questionnaire, respondents are asked “Which of these dietary supplements have you used on a consistent basis during the last 12 months?”. The list of supplements includes nine vitamins/minerals, 16 for power and strength (e.g., beta-alanine and whey), 10 for weight loss (e.g., guarana), nine for endurance (e.g., caffeine), eight for immunity (e.g., antioxidants), six for joint health, and six herbals. Respondents were also allowed to list other dietary supplements that they use which are not listed in the questionnaire. Questionnaire items also asked about the frequency of intake per day during off-season, in-season, and peak week training periods, reasons for supplement use, observations about how the use of supplements affected their performance/activities, and others.

### Measurement of protein intake

The dietary intake data was collected using the Automated Self-Administered 24-Hour (ASA24®) Dietary Assessment Tool, version (2020), developed by the National Cancer Institute, Bethesda, MD [21]. Protein intake was measured from the average of four non-consecutive 24-h dietary recalls (three weekdays and one weekend day). Participants were instructed to report all food, fluids, and dietary supplements they consumed from midnight of the previous day to 11:59 pm of the current day, and were provided the *Participant Quick Start Guide for 24-Hour Recall using ASA24-2018 & ASA24 -2020* instructional materials to assist in completing the dietary recalls [33]. A registered dietitian reviewed and cleaned the data, including addressing known issues, based on the National Cancer Institute’s recommendations [34, 35].

### Sociodemographic and bodybuilder variables

Sociodemographic and bodybuilder variables were collected using the validated online questionnaire. Sociodemographic variables included age, race, ethnicity, height, current weight, educational attainment, employment status, household income from all sources, and exercises as part of a competitor’s training protocol performed for at least 10-min over past 7-days. Exercises and associated metabolic equivalent (MET) values were obtained from the 2011 Compendium of Physical Activities to calculate total MET minutes per week, resistance training MET minutes per week, and aerobic training MET minutes per week [36]. Educational attainment, race and ethnicity questions were based on the 2020 Census [37, 38]. Employment status and household income from all sources items were adapted from the Behavioral Risk Factor Surveillance System 2020 questionnaire [39].

The following competitive women bodybuilder variables were assessed: bodybuilding divisions (i.e., bodybuilding, bikini, fitness, figure, physique, and wellness), competition status (i.e., amateur and professional), total number of years of competition, most recent top competition placing details (i.e., organization name, year, placing, professional/amateur competition, and whether professional status was awarded), competitor type (e.g., designated natural vs. all others), most recent competition weight, lowest off-season weight since last competition, highest off-season weight since last competition, total number of years competing, total number of competitions, time in weeks since the last competition, and weeks remaining till the next competition.

### Data management and statistical analyses

The data collection tools were located on secure websites, with ASA24® having its own researcher site [40] from where collected data can be accessed. In the case of missing data or an unusual value being detected, the researcher attempted to contact the participant to rectify the issue. During the analyses, missing data was handled using multiple-imputation via the expectation–maximization algorithm as described by Graham [41]. Five imputations were used.

The IBM SPSS Statistics Version 28.0 (IBM Corp, Chicago, IL, USA) was used to perform all statistical analyses. All continuous variables were not normally distributed, thus they were 90% winsorized [42]. Descriptive statistics were used to describe the study’s population’s sociodemographic, training, bodybuilding, dietary intake, and supplement use. Repeated measures ANOVA with Sidak multiple comparison testing was used to determine differences between self-reported dietary supplement use across seasons while controlling for current weight, total activity, the number of days require to complete the four 24-h dietary recalls, and the completion of non-consecutive dietary recalls (yes/no).

Multiple linear regressions were performed to assess the relationship among: (a) each behavior (i.e., protein intake and dietary supplement use) with intention and perceived behavioral control, (b) intention with the TPB constructs (i.e., subjective norm, attitude, and perceived behavioral control), (c) the TPB constructs with all their respective belief items for behavioral, normative, and control beliefs. All dependent variables were linear as the standardized residuals were normally distributed and met the assumption of homoscedasticity, plus outliers were not influential as Cook’s distance scores were between -1 to + 1. Pearson’s correlations were utilized to assess the association among: (a) each behavior (i.e., protein intake and dietary supplement use) with intention and perceived behavioral control, (b) intention with the TPB constructs (i.e., subjective norm, attitude, and perceived behavioral control), (c) the TPB constructs with all their respective belief items for behavioral, normative, and control beliefs. In addition, Pearson’s correlations were run to assess potential confounders. Associations between dietary supplement use and all TPB outcome variables were controlled for age, current weight, total activity, the number of days required to complete the four 24-h dietary recalls, total number of years competing, total number of competitions, and the categorical variables completion of non-consecutive dietary recalls (yes or no) and employment status in all multiple linear regressions. Associations between protein intake and all TPB outcome variables were controlled for age, current weight, total activity, the number of days required to complete the four 24-h dietary recalls, and the categorical variables non-consecutive dietary recalls (yes or no), employment status, household income per month, education, and most recent top competition placing organization name in all multiple linear regressions. In addition, energy intake was also adjusted only in the analysis for protein intake.

Individual belief items for each behavioral belief, normative belief, and control belief were TPB main construct predicted scores created by regressing each TPB main construct, i.e., attitude, subjective norm, and perceived behavioral control, on their respective components of each belief item. The components of each belief item contain: (a) the behavioral belief, the outcome evaluation for that belief, and the interaction of those two; (b) the normative referent, the motivation to comply with that referent, and the interaction of those two; and (c) the control belief, perceived power for that control belief and the interaction of those two. An example of one regression equation in the calculation of the predicted score of attitude from the first behavioral belief item is written below with the following acronyms for behavioral belief (BB), outcome evaluation (OE), and their interaction (BB × OE):

$$\mathrm{Attitude }= {\upbeta }_{0} + {\upbeta }_{1} ({\mathrm{BB}}_{1}) + {\upbeta }_{2} ({\mathrm{OE}}_{1}) + {\upbeta }_{3} ({\mathrm{BB}}_{1} \times \mathrm{ O}{\mathrm{E}}_{1})$$

This process would be continued for all the belief items that make up each of the three beliefs, i.e., behavioral, normative, and control. Once the predicted scores from all the belief items were created, each TPB main construct was regressed on each of the predicted scores from the individual belief items contained within behavioral, normative, and control beliefs. For example, using protein intake, attitude was regressed on all 10 predicted scores from each item for behavioral belief. Significance for all analyses was set a priori at *P* < 0.05.

## Results

### Participant characteristics, dietary intake, and dietary supplement use

A total of 171 participants attempted to enroll in the study, but of those 10 did not meet the inclusion criteria and 49 did not complete all four dietary recalls, thus they were excluded. The final analytical dataset was based on 112 (65%). After completion of the questionnaire, it took an average of 12.5 days (SD = 6.0) for participants to complete the four dietary recalls. Sociodemographic, training, and bodybuilding characteristics are presented in Tables 1 and 2. Participants were on average 28.3 years old, 64.5 inches, 129.2 pounds, and most of their in-season competition training comprises of aerobic training (55%). Participants are mainly White (84%), have a bachelor’s degree (51%), employed (56%), have a household income less than $5,999 per month (52%), and were primarily ethnicities other than Hispanic, Latino, or Spanish origin (92%). In terms of bodybuilding characteristics, most of the participants are bikini competitors (73%), amateur status (95%), and placing in the top three (71%) at an NPC competition (83%) between 2019–2020 (81%). Most of the in-season competitors have only competed for a mean of 2.2 years and a mean of three competitions. Energy and macronutrient intakes and dietary supplement use are presented in Table 3. In-season competitors reported consuming a mean protein intake of 155.5 g/d (2.9 g/kg BW/d) and using more dietary supplements during the in-season and off-season than during peak week (*P* < 0.001). In-season dietary supplement use was also significantly (*P* < 0.001) greater than off-season dietary supplement use among in-season competitors.

**Table 1:** Sociodemographic and training characteristics of in-season competitors

Characteristic	Mean	SD
Age, yr	28.3	6.2
Height, cm	163.8	5.8
Current Weight, kg	58.6	6.8
Training^a,b^		
Total activity, MET min/wk	4829.2	2229.9
Aerobic training, MET min/wk	2658.2	1795.1
Resistance training, MET min/wk	1896.2	998.5
	**n**	**%**
Ethnicity		
Not of Hispanic, Latino, or Spanish origin	103	92.0
Mexican, Mexican American, Hispanic, Latino, or Spanish origin	9	8
Race		
White	94	84
Black or African American	6	5
Asian/Pacific Islander	4	4
Other races	8	7
Education^c^		
GED, high school diploma, or less than 1 year of college credit	6	5
One or more years of college credit, no degree	15	13
Associate’s degree	11	10
Bachelor’s degree	57	51
Master’s degree	16	14
Professional degree beyond a bachelor's degree	3	3
Doctorate degree	4	4
Line of work		
Employed for wages	63	56
Self-employed	12	11
Out of work	2	2
Homemaker	2	2
Student	9	8
Retired	1	1
Employed and self-employed	4	4
Student and employed, or self-employed, or all three	15	13
Other combinations	4	4
Household income per month		
$0 to $2,999	27	24
$3,000 to $5,999	31	28
$6,000 to $8,999	16	14
$9,000 to $11,999	10	9
$12,000 or more	28	25

^a^These are the exercises performed only for bodybuilding training and do not include other activities for leisure or work

^b^MET indicates metabolic equivalent

^c^GED indicates general educational development

**Table 2:** Competition characteristics of in-season competitors

Characteristic	n	%
Competitor division		
Bikini	82	73
Bodybuilder	1	1
Figure	16	14
Fitness	2	2
Physique	4	4
Wellness	7	6
Competitor status		
Amateur	101	90.2
Professional	11	10
Never competed	28	25
Most recent top competition placing		
Organization name		
NPC	70	83
IFBB	3	4
WNBF	3	4
NANBF	2	2
OCB	3	4
Other organization	3	4
Year		
2011–2017	9	11
2018	7	8
2019	43	51
2020	25	30
Placing		
1	27	32
2	16	19
3	17	20
4	7	8
5	4	5
6–15	9	11
Did not place	5	6
Type		
Amateur	80	95
Professional	4	5
Professional status obtained?		
Yes	9	11
No	71	89
Self-reported as a "natural" athlete		
Yes	92	82
No	20	18
	**Mean**	**SD**
Most recent competition weight, kg	54.2	4.9
Lowest off-season weight since last competition, kg	58.0	5.4
Highest off-season weight since last competition, kg	64.5	7.0
Total number of years competing	2.2	2.1
Total number of competitions	3.0	3.1
Time to prepare for last competition, wks	18.8	4.8
Time since last competition, wks	50.4	40.8

**Table 3:** Dietary intake and supplement use of in-season competitors

Dietary variable	Mean	SD
Nutrient		
Energy (kcals)	1634.6	462.3
Protein (g)	155.5	38.5
Carbohydrate (g)	156.0	65.8
Fat (g)	60.1	23.0
Alcohol (g)	0	0
Dietary supplement use reported within a season^c,d^		
Off-season	8.8	4.6^a^
In-season	11.2	5.9^b^
Peak week	3.2	2.9
Total	13.0	6.3

^a^Off-season supplement use was significantly greater than peak week supplement use (*P* < 0.001)

^b^In-season supplement use was significantly greater than off-season supplement use and peak week supplement use (*P* < 0.001)

^c^Dietary supplement use during the past 12-months

^d^*P* < 0.05 was considered statistically significant

### Protein intake and the TPB

The standardized regression coefficients (β), 95% confidence intervals (CI), and correlations (*r*) for each belief item, TPB main construct, and intention to consume protein are presented in Table 4 (see Additional file 3). Behavioral beliefs explained 26% of the variance in attitude, while normative beliefs explained 46% of variance in subjective norm. Control beliefs did not significantly explain any of the variance in perceived behavioral control. Attitude, subjective norm, and perceived behavioral control explained 8% of the variance in intention, however only subjective norm was independently positively correlated and predictive of intention. Intention and perceived behavioral control, however, were not predictive of or correlated with protein intake.

Although three outcome beliefs, i.e., “consuming healthier protein with less sugar will help me to get leaner,” “consuming healthier protein with less fat will help me to get leaner,” and “consuming protein will help me to have energy to fuel my workouts,” were positively correlated with attitude, none of the other beliefs were predictive of attitude. Similarly, only two control beliefs, i.e., “having to pay a lot of money for my protein” and “having to travel”, were positively correlated with perceived behavioral control, however only one control belief—"having a meal plan”—was predictive of perceived behavioral control. All of the referent groups except for male bodybuilders and professional competitors were positively correlated with subjective norm. Only three referent others, i.e., trainer/coach, workout partners, and social media influencers, were predictive of subjective norm. Trainer/coach provided the strongest prediction of subjective norm.

### Dietary supplement use and the TPB

The standardized regression coefficients (β), 95% confidence intervals (CI), and correlations (*r*) for each belief item, TPB main construct, and intention to use dietary supplements are presented in Table 5 (see Additional file 4). Intention and perceived behavioral control explained 5% of the variance in dietary supplement use. Intention was also predictive and positively correlated with dietary supplement use. Attitude, subjective norm, and perceived behavioral control explained 38% of the variance in intention with all three constructs being independently positively correlated and predictive of intention. Outcome beliefs explained 53% of the variance in attitude, while referent others explained 59% of the variance in subjective norm. Finally, control beliefs explained 22% of the variance in perceived behavioral control.

All outcome beliefs were positively correlated with attitude, except for two items, i.e., “will be harmful for me during competition preparation” and “will make me dependent on dietary supplements.” Only five outcome beliefs, i.e., “will not be a waste of money,” “will help me to progress further and improve my physique,” “will help me to have enough energy throughout the day,” “will help me to get enough calories, and will help me recover,” were predictive of attitude. All referent others were positively correlated with subjective norm, but only four, i.e., other competitors, competitors educated on dietary supplements, social media influencers, trainer/coach, were predictive of subjective norm. All control beliefs were positively correlated with perceived behavior control, except for one belief, i.e., “I had a dietary supplement company sponsorship.” The three control beliefs, i.e., “dietary supplements were easy to purchase,” “I had enough money to buy dietary supplements,” and “my dietary supplements were more easily available to purchase,” were predictive of perceived behavioral control.

## Discussion

This is the first investigation, to the authors’ knowledge, examining predictors of dietary behaviors in a representative sample of US in-season competitors using the TPB. For protein intake, there was little evidence for the prediction of this behavior using the TPB. Subjective norm, however, in a separate analysis explained (6%) of intent to consume protein when attitude and perceived behavioral control were not controlled. This relationship is only slightly stronger (8%) when intent is regressed on all three TPB variables. It appears, however, the referent others that are important to in-season competitors, in order of most to least influential, were trainer/coach, workout partners, and social media influencers. Although the TPB was not used to examine predictors of dietary intake, Kleiner, Bazzarre, and Litchford [16] indicated that coaches were revered sources of information for women bodybuilders, like the current study. Although subjective norm was correlated with and predictive of intention, it only explained 6% of the variance. This suggests these individuals may only have a small influence over a competitor’s intent to consume protein. There was, however, no evidence for the prediction of attitude, perceived behavioral control or actual protein intake.

Potential reasons for the lack of evidence in the prediction of protein intake by the TPB may be a least partially explained by minimal variation in protein intake and what Ajzen [12] calls a change of mind. Evidence from a study that used the eating restraint subscale score from the Multidimensional Body Self-Relations Questionnaire indicated that women bodybuilding competitors have superb control over their dietary intake [43]. The rigidity of dietary intake is increased during the in-season when competitors prepare for a competition. They engage in short-term energy deficits with high protein intakes in an attempt to enhance satiety and maintain lean body mass [44]. This heighten awareness of dietary intake, particularly the excess consumption of protein, i.e., participants consumed (mean = 2.9 g/kg BW/d) in excess of the recommendations [7], and minimal variation with protein intake (mean = 155.5 g; 95% CI 148.5 – 162.5 g) exhibit what Ajzen [12] highlighted as a restriction of range. A restriction of range indicates the correlations between the intentions or behaviors in participants will be low, which seems to be the case as most of the in-season competitors intend to consume protein and do consume protein. Similar to our study, limited variation in competitor protein intake has also been observed in two recent UK studies [1, 2].

Another potential reason for the limited prediction of protein intake using the TPB is what Ajzen [12] calls a change of mind. As the time interval between when people have expressed their intentions and the actual measurement of the behavior increases, the correlation between them decreases [12]. This decreased correlation between intent and behavior is due to a change in their behavioral, normative, and control beliefs resulting in a change in their original intentions [12]. Intervening events between when participants have expressed their intention and the measurement of the actual behavior could have produced changes in in-season competitors’ beliefs as the average number of days needed to complete the four 24-h dietary recalls was 12.5 days (SD = 6.0) after the completion of the online questionnaire. Whereas dietary supplement use was assessed at the same time as intention within the online questionnaire, thus intent to consume dietary supplements was able to predict dietary supplement use.

In contrast to the lack of evidence for the prediction of protein intake, the prediction of dietary supplement use using the TPB was much more fruitful. Attitude, subjective norm, and perceived behavioral control explained 38% of the variance in intention to use dietary supplements. A study among National Collegiate Athletic Association Division 1 female student athletes indicated attitude, subjective norm, and perceived behavioral control explained 64–66% of variance in intent to consume dietary supplements [15]. Subjective norm was found to be the strongest predictor of intention, [15] whereas our study found attitude and subjective norm to be the strongest predictors of intention. Differences in the sociodemographics and sport characteristics may partially explain this disparity.

In-season competitors believe dietary supplements would not be a waste of money, help them progress and improve their physique, give them energy throughout the day, give them enough calories, and help them to recover. Competition preparation can last four months or more and includes a reduction in energy intake and an increase in physical activity. The change in energy intake and physical activity result in numerous physiologic adaptations that increase hunger and lethargy, while reducing energy expenditure [7]. These undesirable adaptations may partially explain why in-season competitors would find dietary supplements useful during competition preparation. Some dietary supplements, like protein powders, caffeine, creatine, have been recommended to mitigate these adverse physiological changes during competition preparation [7].

In-season competitors also believe other competitors, competitors educated on dietary supplements, social media influencers, and their trainer/coach, are important people that influence their use of dietary supplements. Like protein intake, Kleiner, Bazzarre, and Litchford [16] indicated that coaches were revered sources for dietary supplement information for women bodybuilders. Although other competitors are the strongest predictor of subjective norm, it appears that coaches or trainers and social media influencers are motivators across several dietary behaviors.

Unlike protein intake, control beliefs were predictive of perceived behavioral control for dietary supplement use. In-season competitors were more likely to use dietary supplements if they could purchase them easily, they had enough money to buy dietary supplements, and they were easily available. Having a dietary supplement company sponsorship or the affordability of dietary supplements, however, were not predictors of perceived behavioral control. A lack of significance for those two control beliefs are surprising given 52% had a household income of less than $6,000 and having enough money to buy dietary supplements was significant. In contrast, dietary supplements are readily available and easy to purchase around the globe due to the proliferation of the internet [45]. This may partially explain why perceived behavioral control was the weakest predictor of intent to consume dietary supplements.

Although intention to use dietary supplements was predictive of dietary supplement use the amount of variance explained was only 5%. Ajzen [12] indicates that people do not always perform the behavior even if intentions to do so are strong. In fact, Ajzen [12] indicates that intention to perform a behavior often only accounts for about 25% of variance in the execution of the behavior [46]. One factor that may have played a role has been called a lack of scale compatibility [12]. This factor indicates that intention to perform a behavior, e.g., I intend to consume dietary supplements during competition preparation, is measured in means of probabilistic scales, i.e., extremely likely—extremely unlikely, I definitely will not—I definitely will, and strongly disagree—strongly agree, while behavior is determined by frequency, i.e., how many dietary supplements competitors consume during the in-season [12]. The discrepancy in scale compatibility in the current study may be one reason why the prediction and correlation of dietary supplement use by intention was low.

The limited evidence for the prediction of protein intake and small amount of variance explaining dietary supplement use by intention also may be due to the in-season competitors being mostly bikini (73%). Bikini competitors are required to have the least amount of muscular definition and development out of all of the other competitor divisions [47], thus they may have different protein intake and supplement use behaviors and motivators. Post-hoc ANCOVA analyses were used to assess the differences between bikini competitors (*n* = 86) and all other competitor’s (*n* = 31) protein intake and dietary supplement use. Bikini competitors consumed significantly less protein and used significantly fewer dietary supplements during the in-season compared to all other competitors as presented in Supplementary Table 3 (see Additional file 5).

Multiple linear regressions were performed to assess the relationship among: (a) each behavior (i.e., protein intake and dietary supplement use) with intention and perceived behavioral control, (b) intention with the TPB constructs (i.e., subjective norm, attitude, and perceived behavioral control), (c) the TPB constructs with all their respective belief items for behavioral, normative, and control beliefs for bikini competitors and all other competitors. The results of the regression analyses for protein intake indicated that behavioral beliefs explained 27% of the variance in attitude, while normative beliefs explained 50% of variance in subjective norm in bikini competitors. Unique only to bikini competitors, the referent other “athletes” were significantly predictive of subjective norm (β = 0.24, *p* < 0.048). Control beliefs did not significantly explain any of the variance in perceived behavioral control, nor was intention and perceived behavioral control predictive of protein intake. These results were like those for all in-season competitors. Contrary to all in-season competitors, attitude, subjective norm, and perceived behavioral control together did not explain any of the variance in intention in bikini competitors.

The results of the regression analyses for protein intake did not significantly explain any of the variance in attitude, subjective norm, or perceived behavioral control in all other competitors. Unique only to all other competitors the behavioral belief, “will help me to build muscle”, was predictive of attitude (β = -0.49, *p* < 0.049). Like the results for all in-season competitors and bikini competitors, intention and perceived behavior control was not predictive of protein intake. Contrary to the bikini competitors, attitude, subjective norm, and perceived behavioral control together were predictive of intention (β = 0.32, *p* < 0.04) for all other competitors. The previously aforementioned results are like those for all in-season competitors, however, unlike all in-season competitors attitude (not subjective norm) was the only significant predictor of intention (β = 0.75, *p* < 0.002).

The results of the regression analyses for dietary supplement use in bikini competitors indicated that behavioral beliefs explained 56% of the variance in attitude, normative beliefs explained 65% of variance in subjective norm, and control beliefs explained 28% of the variance in perceived behavioral control. These results were like those for all in-season competitors. Contrary to all in-season competitors, the referent other “professional competitors” were significantly predictive of subjective norm (β = 0.33, *p* < 0.03) and the control belief “dietary supplements were affordable” was significantly predictive of perceived behavioral control (β = -0.29, *p* < 0.045) in bikini competitors. Also, like all in-season competitors, attitude, subjective norm, and perceived behavioral control together explained 28% of the variance in intention, while only attitude (β = 0.42, *p* < 0.001) and subjective norm (β = 0.43, *p* < 0.001) were independently predictive of intention. Contrary to all in-season competitors, perceived behavioral control and intention together did not explain any of the variance in dietary supplement use.

The results of the regression analyses for dietary supplement use in all other competitors did not significantly explain any of the variance in attitude, subjective norm, perceived behavioral control, intention, or the behavior itself. Interestingly, like the bikini competitors, the control belief “dietary supplements were affordable” was a significant predictor of perceived behavioral control (β = -0.49, *p* < 0.04) in all other competitors. Also, like all in-season competitors, yet unlike bikini competitors, perceived behavioral control was a significant independent predictor of intention (β = 0.51, *p* < 0.01).

The results of these post hoc analyses indicate that there are different protein and supplement behaviors between the in-season bikini competitors and all other in-season competitors. In addition, the motivators for these behaviors seem to differ slightly too, albeit the evidence is limited. The sample size for all other competitors, however, was small (*n* = 31). A larger sample of each category of in-season competitors will be needed to have enough power to adequately assess these behaviors and their motivators. It is worth noting that the development of our validated survey included the input from a diverse set of women bodybuilding competitors, i.e., bikini, figure, physique, and wellness, thus our survey should be appropriate for use across these different competitor divisions. Regardless, the limited results from these post hoc analyses warrant further investigation.

There are several strengths and a few limitations to the present study. To the authors’ knowledge this is one of largest dietary intake and behavior studies, with the inclusion of dietary supplements, in a representative sample of US in-season competitors. Generalizability of the study results is limited to only these behaviors and in-season competitors, including those with their sociodemographic characteristics, i.e., ethnicity, race, educational attainment, employment status, and monthly household income. This was also the first time a validated questionnaire was designed to ascertain sociodemographic, bodybuilder, dietary supplement, and TPB variables in in-season competitors. It should be noted; however, that all data was self-reported and has the potential to contain reporting errors that could affect dietary intake estimates and biometric data. Although inaccurate body weights would affect the energy and macronutrient calculations relative to body weight, it is common practice for bodybuilders to regularly weigh themselves [2] and track their dietary intake and training regimes [48].

Data collection took place during the height of the COVID-19 pandemic making it plausible that there was a history threat to internal validity [49]. The pandemic negatively affected all areas of life, e.g., gym access and food supply chain issues [50, 51]. In one study, 33.8% of participants indicated that gyms being closed was a major barrier to engaging in their regular exercise routines [51]. Similarly, food shortages reduced access to a variety of foods, particularly protein-containing foods, like meat, eggs, and pork [50]. In addition, the increased cost of food altered food buying behaviors [50]. Also, how people coped with the pandemic altered food purchasing behaviors, e.g., purchasing more healthy foods or purchasing more unhealthy foods [52]. It is unclear how the pandemic truly affected the dietary and supplement intake behaviors and training regimes of our study’s competitive women bodybuilders, as those specifics were not assessed. During some of the text and email study completion reminders, however, a few in-season competitors mentioned their workout routines changed due to a limited access to their gym, while others indicated they had a longer in-season due to the cancelation of bodybuilding shows. The mean protein intake for in-season competitors from the current study, however, were like the protein intake of other in-season competitors from several recent studies prior to the pandemic [1–6, 53]. Similarly, dietary supplement usage was reported by 100% of in-season competitors in several studies, which is congruent with our findings [6, 23].

Several techniques were used to increase the accuracy of the dietary intake data. To be within 10% of the 365 day average intake for protein, four dietary recalls are recommended and used in the present study [54]. In addition, the ASA24® is a valid tool for collecting dietary intake data for research [55]. The ASA24® employs the use of the multiple-pass method which increases the accuracy of estimating self-report dietary intake data, particularly in lean women [56, 57]. Finally, the expertise of a registered dietitian was employed in the dietary intake and supplement use data collection and analyses.

Finally, it should be noted, the authors strictly adhered to methods outlined by Ajzen [25] in constructing and administering of the TPB questionnaire. Regardless, examining the association between intent to perform a behavior and the execution of that behavior at a later date is problematic. Intent to perform a behavior appears to only account for about 25% of the variance in that behavior [12, 46]. Ajzen [12] outlines numerous factors that may impede our understanding of the relationship between intention and the execution of the behavior, several of which, i.e., change of mind, restriction of range, and lack of compatibility, potentially affected our understanding the two behaviors examined in the present study.

## Conclusion

While these findings are informative and provide insight into the influential beliefs that help to determine protein intake and dietary supplement use for in-season competitors, additional research is still needed to enhance our understanding of how these dietary behaviors are directed in this population. Of particular interest would be to assess whether the lack of prediction of protein intake from the TPB was due to the minimal variation in protein intake or the time interval between assessing the TPB and dietary protein intake. In addition, correlations are suggestive of a relationship between these two behaviors and the TPB, however, an experimental study would be necessary to test these associations. Finally, as illustrated in Table 5 (see Additional file 4), intention only explained 5% of the variance in dietary supplement use, it is plausible that other unknown variables may affect these behaviors.

In summary, in-season competitors in this study have a higher than recommended protein intake and possibly consume dangerous dietary supplements during the in-season. Thus, understanding the drivers of protein intake and dietary supplement use are invaluable resources to help guide health practitioners to develop effective interventions. By identifying the key influences of these behaviors, we can develop strategies to help shape in-season competitors’ protein intake and dietary supplement use. Although the evidence to recommend strategies for modifying protein intake in in-season competitors is inconclusive, strategies to modify dietary supplement consumption in this population would benefit from aligning health education messages with the significant outcome beliefs, i.e., will not be a waste of money, will help me to progress further and improve my physique, will help me to have enough energy throughout the day, will help me to get enough calories, and will help me recover, in that they were significant predictors of attitude. Similarly, health practitioners could benefit from collaborating with referent others, i.e., other competitors, competitors educated on dietary supplements, social media influencers, trainer/coach, to help align health education messages to influence healthier dietary supplement consumption patterns for in-season competitors. Finally, if health practitioners can help to change dietary supplement intake patterns in in-season competitors this may also influence the dietary supplement choices of bodybuilding adherents.

### Supplementary Information

**Additional file 1: Supplementary Table 1.** The TPB main construct questions for protein intake for in-season competitors.

**Additional file 2: Supplementary Table 2.** The TPB main construct questions for dietary supplement use for in-season competitors.

**Additional file 3: Table 4.** The predictors of protein intake for in-season competitors using the TPB.

**Additional file 4: Table 5.** The predictors of dietary supplement use for in-season competitors using the TPB.

**Additional file 5: Supplementary Table 3.** Dietary intake and supplement use of in-season bikini and all other competitor divisions.

## Data Availability

The datasets generated during and/or analyzed during the current study are not publicly available due the nature of participant confidentiality but deidentified data are available from the corresponding author (JEH) on reasonable request.

## Abbreviations

ASA24: Automated self-administered 24-hour dietary assessment tool
BB: Behavioral belief
BW: Body weight
CVI: Content validity indices
MET: Metabolic equivalent
OE: Outcome evaluation
TPB: The theory of planned behavior
